# Feasibility of a Novel Autoinflation Device to Treat Pediatric Otitis Media With Effusion At‐Home

**DOI:** 10.1002/oto2.70128

**Published:** 2025-05-14

**Authors:** Maria‐Jose Soto, Nanki Hura, Intan Oldakowska, Matthew Oldakowski, Paul Bumbak, Peter Luke Santa Maria

**Affiliations:** ^1^ Centre for Medical Research at the University of Western Australia Crawley Western Australia Australia; ^2^ Department of Otolaryngology University of Pittsburgh Pittsburgh Pennsylvania USA; ^3^ Department of Otolaryngology, Head and Neck Surgery Perth Children's Hospital Nedlands Western Australia Australia

**Keywords:** autoinflation, eustachian tube dysfunction, hearing loss, otitis media with effusion, pediatric

## Abstract

**Objective:**

Otitis media with effusion (OME) affects 90% of children before school age, with current guidelines recommending tympanostomy tubes for persistent OME and hearing loss after 3 months of “Watchful Waiting,” due to the risk of complications including long‐term conductive hearing loss. Current nonsurgical treatment options are limited. This study evaluates the feasibility of at‐home use of a novel prototype autoinflation device for children with OME or eustachian tube dysfunction.

**Study Design:**

Single‐arm cohort study.

**Setting:**

Pediatric otolaryngology private clinic.

**Methods:**

Children aged 1 to 12 years with OME were recruited from a pediatric otolaryngology clinic and asked to use the device twice daily for 4 weeks. Baseline audiometry and tympanometry were performed at recruitment, with subsequent tympanometry testing after first use and at 2, 4, 8, and 12 weeks. Audiometry testing was repeated at week 4. Compliance was tracked using an App.

**Results:**

Twenty‐one patients were included (average age: 5.1 years, range: 2‐12 years). After a single session, 86% of patients had middle ear pressure improvement in at least one ear, which maintained stable at 83% at week 4. Additionally, 86% of patients with hearing loss exhibited improvement at 4 weeks, where the average pure tone average in ears with baseline hearing loss improved from 26.8 to 18.9 dB HL.

**Conclusion:**

These results demonstrate significant improvement in both tympanometry and audiometry after 4 weeks of device use. This indicates a strong potential benefit in regular ventilation of the middle ear for pediatric patients with OME undergoing “Watchful Waiting.”

Otitis media with effusion (OME) is defined by the presence of fluid in the middle ear without signs or symptoms of an acute ear infection and affects around 90% of children before school age.^1^, ^2^ In the United States, about 2.2 million episodes of OME are diagnosed annually, costing $4 billion.^1^, ^3^ OME frequently occurs as a sequelae of acute otitis media and/or in the setting of dilatory eustachian tube dysfunction (ETD). Although most episodes of OME resolve spontaneously, at least 25% of episodes persist for 3 months or longer.^4^ When left untreated, OME can result in otalgia, sleep disruption, balance symptoms, as well as chronic conductive hearing loss, which can lead to language and speech delays, poor school performance, behavioral problems, and overall reduced quality of life.^1^, ^5^, ^6^, ^7^

Current American Academy of Otolaryngology–Head and Neck Surgery (AAO‐HNS) guidelines recommend a “Watchful Waiting” approach for a 3‐month period for patients with OME who are not at increased risk for speech, language, or learning issues.^1^ After this period, patients with persistent unilateral or bilateral OME, with documented hearing difficulties or other symptoms attributable to OME, are recommended surgical intervention with myringotomy with tympanostomy tube insertion.^1^, ^5^ Though a short and minimally invasive procedure, tympanostomy tube placement is not without surgical risks or expense to the health care system, and may be a source of distress for patient families reluctant to undergo anesthesia.^8^

Consequently, nonsurgical techniques have emerged to conservatively manage OME during the “Watchful Waiting” period. Most of these methods are based on either the Valsalva maneuver, which requires forced exhalation with a closed mouth and nose, or the Politzer method, which requires air to be delivered into the nose during swallowing. Although these devices have shown some beneficial short‐term effects, they are limited by multiple factors, including an inability to objectively confirm when a treatment has been successfully delivered, discomfort due to overpressurization, difficulty with use especially in patients younger than 4 years, and low compliance in younger populations.^9^, ^10^, ^11^, ^12^, ^13^

In this study, a novel prototype autoinflation device, designed to enhance accessibility for younger patient populations, was introduced. The aim of this study was to investigate the feasibility of this autoinflation device for at‐home management of OME in pediatric patients, as well as assess the effect of regular ventilation of the middle ear on tympanometry and audiometry outcomes in these patients.

## Methods

### Study Design

A single‐arm cohort study was conducted from October 2023 to May 2024 following Curtin University Human Research Ethics Committee approval (HRE2020‐0526). All patients were recruited at a single practice, Paediatric ENT Services clinic in Western Australia, where they were being evaluated for possible tympanostomy tube placement by a pediatric otolaryngologist. Inclusion criteria were English‐speaking children aged 1 to 12 years with either a type B or C tympanogram in at least one ear, a confirmed diagnosis of OME by an otolaryngologist, and the ability to use the device after training by the research assistant. Exclusion criteria were patients with craniofacial abnormalities, active acute upper respiratory or middle ear infections, tympanostomy tubes currently in situ, history of velopharyngeal insufficiency, or history of swallowing dysfunction. Written consent was obtained from the parents or legal guardians of all participants at the time of enrollment.

### Autoinflation Device

**Figure 1:**
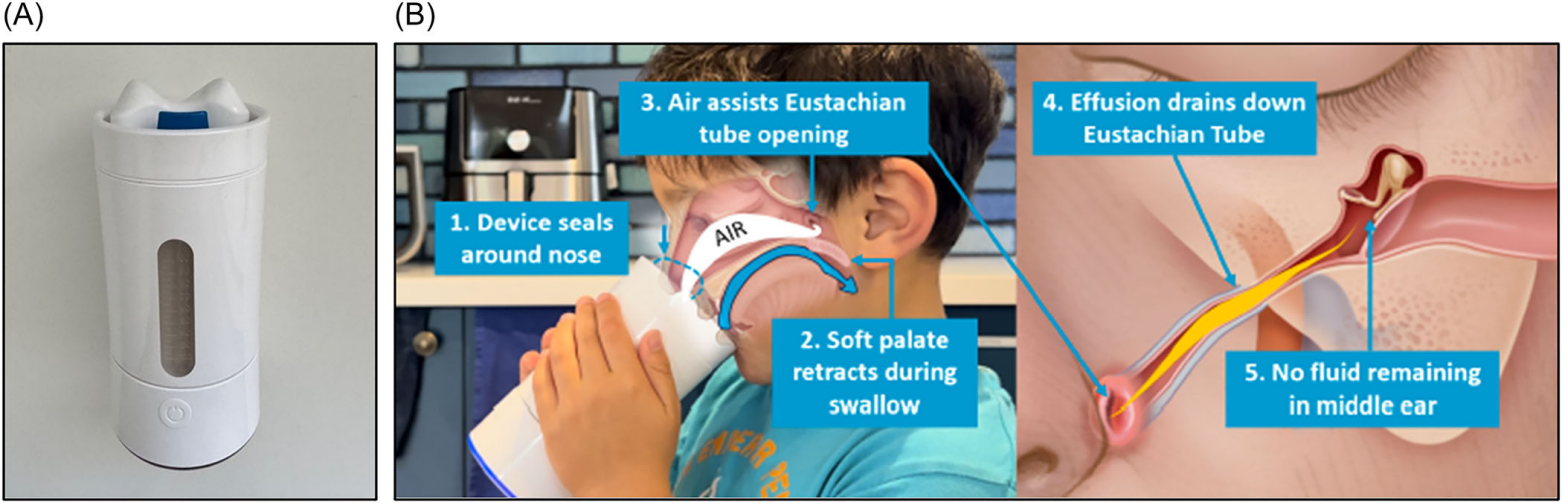
Prototype device used in the study. (A) The prototype device. (B) Mechanism of action of prototype device (illustration by Chris Gralapp).^14^

A novel prototype autoinflation device was used in this study (Figure 1). The device, shaped like a drinking bottle, had a top consisting of a nasal mask and a drinking spout. When the patient drinks from the cup, their nose engages with the nasal mask, which creates a tight seal over the nares. The device uses low‐pressure, continuous nasal airflow to detect when the patient's soft palate retracts during initiation of the oropharyngeal swallow phase. In coordination with the patient's swallow, a puff of air is delivered into the nasal cavity. This puff of air is controlled using an internal pressure sensor and micro‐controlled air pump up to a set‐point of 1 psi. Once the puff of air was delivered and a successful treatment had been achieved (>0.5 psi for >100 ms), the LED light at the base of the device turned green.

The device was paired with a phone application, which logged and displayed the number of successful treatments the patient achieved over the trial period. The app also contained a rocket ship game that gamified device use for pediatric patients.

### Data Collection

Upon enrollment, patient families were instructed on how to use the device and phone application by the research assistant. Participants underwent baseline audiometry and tympanometry testing at the first visit. Afterward, patients were encouraged to use the device for a treatment session in the clinic, with one treatment session defined as five successful administrations of the controlled puff of air. Tympanometry was repeated after this session. Patients then took the device home for 4 weeks and were asked to perform two sessions daily, with a goal of 10 successful treatments per day.

Patients had follow‐up appointments at 2, 4, 8, and 12 weeks. Tympanometry assessments were conducted at all follow‐up appointments. Tympanometry outcomes included the tympanogram classification (type A, B, or C), based on the modified Jerger classification, and middle ear pressure (MEP).^15^ A type A tympanogram was defined as having a peak identified within ±100 daPa, and a type C tympanogram was defined as having a peak identified below −100 daPa. A type B tympanogram does not have an identifiable peak. An interval audiometric assessment was performed at the 4‐week follow‐up. For audiometry, the pure tone average (PTA) was calculated by averaging the decibels hearing loss (dB HL) for the 500, 1000, 2000, and 4000 Hz frequencies. All tympanometry and audiometry testing was conducted by an audiologist.

### Outcomes

The primary outcomes were tympanometry and audiometry results. For tympanometry, a difference in MEP of 50 daPa or greater from baseline was defined as a “change”; this accounted for variation in device measurement, which can be ±10 daPa. For audiometry, an improvement was defined as a change of at least 10 dB HL in any frequency with hearing loss or change into normal hearing based on AAO‐HNS classification (0‐20 dB HL PTA).^1^ Only ears with documented hearing thresholds on the baseline audiogram that were >20 dB HL PTA were analyzed for subsequent audiometric improvement using these definitions. A secondary outcome was compliance, defined by the number of days the patient successfully used the prototype device for at least a single treatment session over the total number of treatment days. Compliance was tracked through a pressure sensor in the device.

### Statistical Analysis

Two‐tailed paired *t* tests were used, with normality of the data verified using a Shapiro‐Wilk test. A Wilcoxon signed‐rank test was used to assess differences in any data that were not normally distributed. For statistical analysis of tympanogram measurements where no peak value was recorded, typical of type B tympanograms, a value of −450 daPa was assigned. This value is consistent with previous studies that used either the same or a similar value given that the tympanometer measures down to −400 daPa.^11^, ^12^

## Results

### Demographics

**Figure 2:**
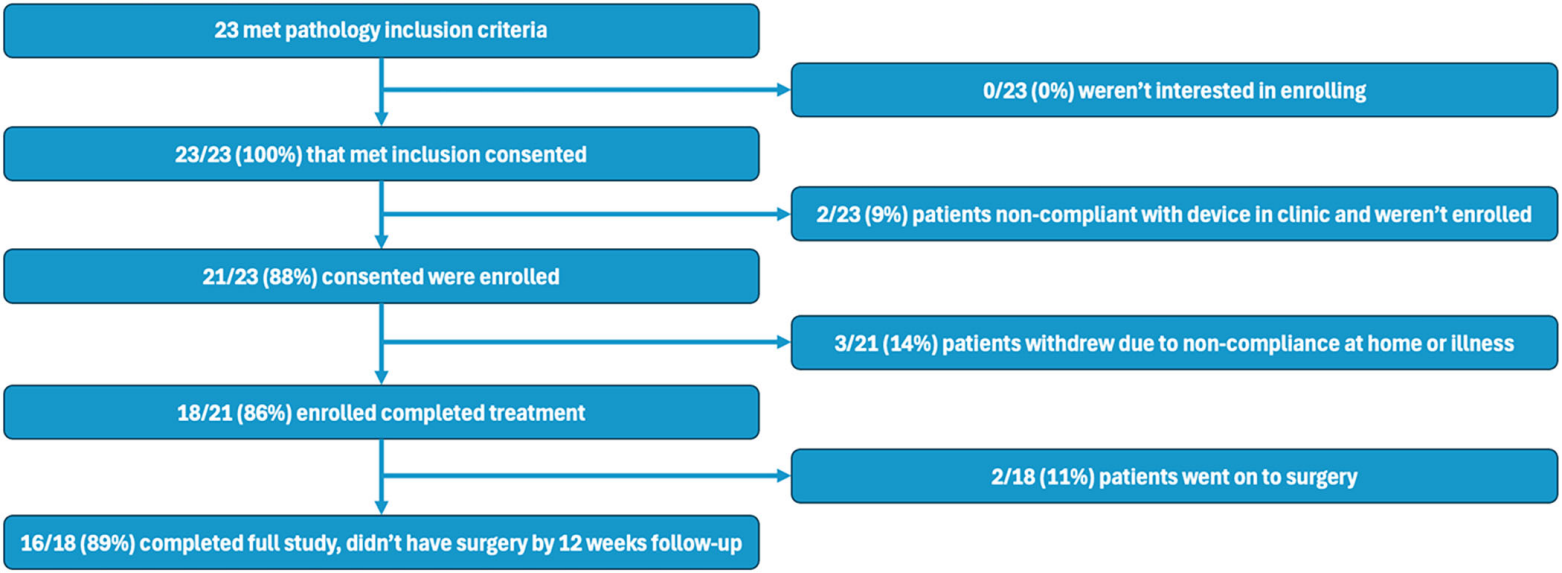
Patient numbers throughout the study. The participation and compliance of patients at different stages of the study.

Twenty‐three patients were consented and assessed for eligibility, with two patients unable to successfully use the device after training (ages 1 year 8 months and 2 years 7 months) and therefore not included. A total of 21 patients were included (11 females, 52%; average age: 5.1 years; range: 2‐12 years) (Figure 2). Of 42 total ears, 50% had type B tympanograms (n = 21) and 50% had type C tympanograms (n = 21) on initial tympanometry assessment. Three of 21 patients (14.3%) withdrew from the study before completion of the 4‐week study period: 1 withdrew due to illness unrelated to the study, and the remaining 2 withdrew as their parents were unable to convince them to use the device at home. Overall, no adverse events or complications were reported during the study, nor since study completion to date.

### Tympanometry Results

After a single treatment session, 86% of patients (18/21) and 64% of ears (27/42) had improvement in MEP (Table 1). In this same session, 14% and 29% of type B ears converted to a type A or C tympanogram, respectively. Of ears that had a baseline type C tympanogram, 52% resolved to a type A tympanogram.

**Table 1:** Tympanometry Results^a^

	Day 1	Week 2	Week 4	Week 8	Week 12
Patient improvement in MEP at least one ear	86% (18/21)	78% (14/18)	83% (15/18)	86% (12/14)	63% (10/16)
Ears with improvement in MEP	64% (27/42)	67% (24/36)	61% (22/36)	68% (19/28)	52% (16/31)
Type B ears converted to type A	14% (3/21)	17% (3/18)	18% (3/17)	17% (2/12)	8% (1/13)
Type B ears converted to type C	29% (6/21)	56% (10/18)	35% (6/17)	33% (4/12)	38% (5/13)
Type C ears converted to type A	52% (11/21)	50% (9/18)	53% (10/19)	69% (11/16)	39% (7/18)

Abbreviation: MEP, middle ear pressure.

^a^ Differences in sample sizes are due to patients who withdrew or did not attend their appointments on the day.

By week 2, 78% (14/18) of patients and 67% (24/36) of ears had an improvement in MEP, with 17% and 56% of ears with a baseline type B tympanogram converting to a type A or C tympanogram, respectively. Additionally, 50% of type C ears resolved to a type A tympanogram.

By week 4, 83% (15/18) of patients and 61% (22/36) of ears had improvement in MEP, with 18% and 35% of ears with a baseline type B tympanogram now displaying a type A or C tympanogram, respectively. In addition, 53% of type C ears resolved to a type A tympanogram. After discontinuing device use at week 4, the proportion of patients with improvement in MEP relative to baseline decreased to 86% and 63% at weeks 8 and 12, respectively. Of the 15 patients that showed MEP improvement at week 4, 5 (33%) had worsened MEP by week 12. Four of these 5 patients (80%) reported sickness during the post‐treatment study period, compared to 5 of the 10 patients (50%) reporting sickness in the group of patients that maintained MEP improvement at week 12.

### Audiometric Results

**Figure 3:**
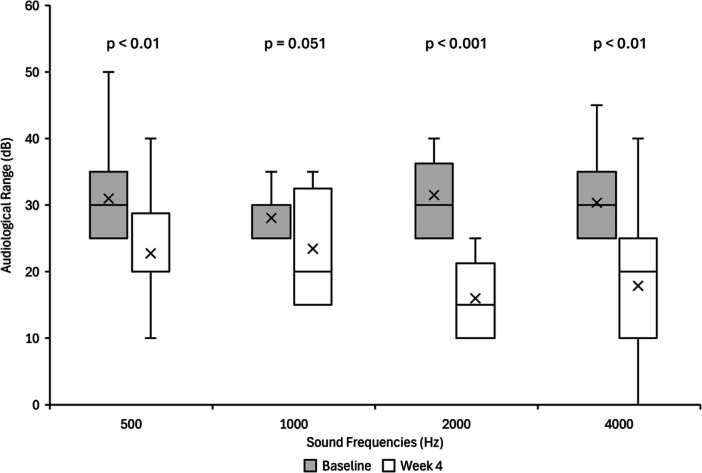
Audiological range‐hearing loss ears. Frequencies with initial hearing loss are compared. Comparison at 500 Hz (n = 20), 1000 Hz (n = 13), 2000 Hz (n = 10), 4000 Hz (n = 14).

The baseline PTA of all ears with hearing loss was 26.8 dB HL (Table 2). The baseline PTA of ears with type B tympanograms and type C tympanograms was 28.4 and 24.2 dB HL, respectively. After 4 weeks of device use, the average PTA in all ears with hearing loss improved from 26.8 to 18.9 dB HL, and the average PTA for type B and type C tympanograms improved by 6.8 and 9.4 dB HL, respectively. Overall, 85% (17/20) of ears and 86% (12/14) of patients with initial PTA hearing loss exhibited improvement at 4 weeks relative to baseline. In total, 60% (12/20) of ears with initial PTA hearing loss returned to normal hearing at 4 weeks. The average PTA improvement per ear with initial hearing loss, as calculated across all measured frequencies, including frequencies with thresholds within the normal hearing range at baseline, was 7.9 dB HL. This value was slightly higher for children older than 4 years (8.4 dB HL) compared to children younger than 4 years (7.0 dB HL), though this difference was not statistically significant (*P* = .685). The average dB HL improvement in ears with initial PTA hearing loss was 14.5 dB HL, measured across only the frequencies with hearing loss for each ear. Significant improvements were observed for three of the frequencies by week 4, when assessing ears with hearing loss in specific frequencies from baseline (Figure 3).

**Table 2:** Pure Tone Average (PTA) Audiometry Results

	Baseline	Week 4
Average PTA in ears with hearing loss, dB HL	26.8 (n = 20)	18.9 (n = 20)
Average PTA in type B ears with hearing loss, dB HL	28.4 (n = 12)	21.6 (n = 12)
Average PTA in type C ears with hearing loss, dB HL	24.2 (n = 8)	14.8 (n = 8)

### Compliance

Three of 21 patients (14.3%) withdrew from the study before completion of the 4‐week study period. Excluding those patients, the average compliance with the device across the 4‐week period was 78.6% (range: 32%‐100%). Patients with improvement in MEP by week 4 (15/18) had an average device compliance of 80% versus patients who did not have improvement in MEP by week 4 (3/18), who had an average device compliance of 70% (*P* = .511).

### Avoidance of Tympanostomy Tube Placement

Sixteen of 18 patients (89%) that completed the full 4‐week study did not proceed with tympanostomy tube placement at the end of the study, whereas 2 patients (11%) were recommended to proceed with tympanostomy tube placement by the pediatric otolaryngologist at the 4‐week visit and consequently withdrew from the remainder of the trial. These two patients had at least one ear without significant improvement in audiometry and tympanometry outcomes, and one of the patients had a recorded compliance of only 32% throughout the 4‐week treatment period.

## Discussion

Tympanostomy tube surgery is one of the most commonly performed surgical procedures, with more than 700,000 performed annually in the United States.^16^ Despite its commonality, tympanostomy tube placement is not without risks, including tube‐associated otorrhea (26%), chronic tympanic membrane perforation (2%), and cholesteatoma (0.5%), and may be a source of stress for parents with strong desires to avoid surgery for their children.^16^ In many areas, limited access to trained otolaryngologists can also lead to prolonged waiting times of up to several months for tympanostomy tube placement.^17^ As untreated OME can have detrimental implications for language/speech development, school performance, and quality of life, there is an unmet need for a nonsurgical method of managing OME.^1^, ^5^, ^6^, ^7^

There has been increasing interest in using autoinflation of the eustachian tube as a nonsurgical intervention for pediatric OME, especially during the “Watchful Waiting” period. Autoinflation devices have largely been deemed as safe and low‐cost, though, with a key limitation of decreased treatment adherence in the younger pediatric population.^1^, ^18^ Studies evaluating existing autoinflation devices have only demonstrated improved outcomes in older children, where Banigo et al reported a reduction in tympanostomy tube surgery rates in children from 79% in controls to 53% after 7 weeks of EarPopper® use.^13^ Williamson et al also demonstrated a significant increase in the number of patients with normal tympanograms after 1 and 3 months of Otovent® use, relative to controls.^19^ Due to doubts regarding autoinflation performance reliability in young children, both studies had an inclusion criterion of 4 years or above.^13^, ^19^ However, OME most commonly affects children between ages 2 and 4, with tympanostomy tube surgery being performed 2.5 times more in children aged 2 to 3 years than children aged 4 to 5 years.^16^, ^20^ Hence, a need exists for a device to provide autoinflation of the eustachian tube in a comfortable and convenient form factor, especially for children younger than 4 years.

This study demonstrates that the novel prototype device can successfully ventilate the middle ear with the potential to help in the treatment of OME, especially for younger pediatric patients. The youngest patient successfully treated in this cohort was 2 years of age, and 24% of included patients were younger than 4 years. Overall, the mean improvement in hearing levels per ear across all frequencies was 7.9 dB HL, which is comparable to that reported from tympanostomy tube placement in both a Cochrane meta‐analysis review (5.2 dB HL) and the AAO‐HNS guidelines (5‐12 dB HL).^16^, ^21^ There was no statistically significant difference in the overall PTA improvement in the younger than 4 years and older than 4 years subgroups. Tympanometry results also reflected significant improvement in MEP for most patients (86%), with approximately 50% of patients converting from a type B to type A or C tympanogram, as well as from a type C to a type A tympanogram, after 4 weeks of device use. In addition, of all the patients who successfully completed a 4‐week use period, 89% avoided being recommended for surgery.

A minority of patients (33%) with improvement in tympanometry results during the study period had subsequent worsening after discontinuation of device use. This may be due to the recurrence of infection, as 80% of these patients reported sickness during this period, compared to 50% of patients in the group that did not decline following device discontinuation. Nonetheless, this draws attention to a select group of patients who could potentially benefit from increased, prolonged, or rescue use of the autoinflation device. Moreover, there were 2 patients that were recommended tympanostomy tube surgery after a lack of improvement in at least one ear during the study period. These patients experienced either partial benefit or may not have had sufficient use of the device to effect change. This reflects a second, important group of patients who may not improve during the “Watchful Waiting” period from autoinflation device use at all. With investigation of this subgroup in future studies, it could be recommended that patients who never respond to the autoinflation device are expedited to tympanostomy tube placement.

A significant challenge to autoinflation device implementation for pediatric patients is ensuring treatment adherence or compliance. Previous autoinflation studies reported compliance based on patient self‐reported device usage, with compliance rates ranging from 24% to 94%.^13^, ^19^ However, as patient self‐reporting is a qualitative measure, it is not evident whether those devices were truly used by the patients or if patients even achieved the required treatment pressure. In this study, a pressure sensor within the device measured how often patients reached the required pressure for a successful treatment, demonstrating an average compliance of 78.6% across the study period. Of the two patients ultimately recommended for tympanostomy tube surgery, one had a compliance of only 32% throughout the study period, suggesting that they may not have used the device sufficiently to get a significant benefit. The tympanometry outcomes after initial use, at 2 weeks, and at 4 weeks, also suggest that improvements may occur earlier than previously reported, highlighting the importance of more meaningful treatment sessions.^11^, ^12^, ^13^ In this study, the device was also paired with an app to gamify and incentivize device use, though, the proportion of patients who utilized this feature was not recorded. Of the 23 patients consented to the study, 5 patients did not complete the study, of which 1 had an unrelated illness, 2 were unable to use the device in the clinic, and 2 were unable to be convinced to use the device at home by their parents. This indicates that most children could use the device immediately.

As this was a feasibility study, there was no control or comparison group. Though this will be a goal of a future study. Limitations to this study include its small sample size and limited audiometric follow‐up. Additional clinical studies will aim to establish the efficacy of this novel autoinflation device as a treatment option in a broader population with a greater range of hearing loss severity. An additional area of study is to investigate whether hearing begins to improve earlier than 4 weeks, given the significant improvements in MEP seen in this study at the 2‐week follow‐up.

## Conclusion

The results of this study demonstrate the feasibility of this novel autoinflation device for children with OME. For children who are in the “Watchful Waiting” period or on the waitlist for surgery, autoinflation of the eustachian tube could provide some relief to patient symptoms and has potential to help children avoid surgery.

## Author Contributions

**Maria‐Jose Soto**, study design, data collection, data analysis, manuscript writing, and review; **Nanki Hura**, data analysis, manuscript writing, and review; **Intan Oldakowska**, study design, data collection, data analysis, manuscript writing, and review; **Matthew Oldakowski**, study design, data analysis, manuscript writing, and review; **Paul Bumbak**, study design, data collection, manuscript writing, and review; **Peter Santa Maria**, study design, data analysis, manuscript writing, and review.

## Disclosures

### Competing interests

Intan Oldakowska, Matthew Oldakowski, and Peter Santa Maria are listed as inventors and shareholders in the company developing and commercializing the device under evaluation. Maria‐Jose Soto is an employee of the company.

### Funding source

This study was funded by the Western Australia Child Health Research Fund.
